# A comparison of PCR and ELISA methods to detect different stages of *Plasmodium vivax* in *Anopheles arabiensis*

**DOI:** 10.1186/s13071-021-04976-z

**Published:** 2021-09-15

**Authors:** Allison L. Hendershot, Endashaw Esayas, Alice C. Sutcliffe, Seth R. Irish, Endalamaw Gadisa, Fitsum G. Tadesse, Neil F. Lobo

**Affiliations:** 1 Eck Institute for Global Health, University of Notre Dame, Notre Dame, IN USA; 2 Malaria and Neglected Tropical Diseases Research Directorate, Armauer Hansen Research Institute, Addis Ababa, Ethiopia; 3 Entomology Branch, Division of Parasitic Diseases and Malaria, Centers for Disease Control and Prevention, Atlanta, GA USA; 4 President’s Malaria Initiative, Bureau for Global Health, Office of Infectious Disease, United States Agency for International Development, Washington DC, USA; 5 Institute of Biotechnology, Addis Ababa University, Addis Ababa, Ethiopia; 6 Radboud University Medical Center, Nijmegen, The Netherlands

**Keywords:** CSP ELISA, COX-I PCR, *Plasmodium*, Infectious mosquitoes, Wild mosquitoes, Infectious reservoir

## Abstract

**Background:**

In characterizing malaria epidemiology, measuring mosquito infectiousness informs the entomological inoculation rate, an important metric of malaria transmission. PCR-based methods have been touted as more sensitive than the current “gold-standard” circumsporozoite (CSP) ELISA. Wider application of PCR-based methods has been limited by lack of specificity for the infectious sporozoite stage. We compared a PCR method for detecting the parasite’s mitochondrial (mt) cytochrome oxidase I (*COX-I*) gene with ELISA for detecting circumsporozoite protein for identification of different life stages of the parasite during development within a mosquito.

**Methods:**

A PCR-based method targeting the *Plasmodium* mt *COX-I* gene was compared with the CSP ELISA method to assess infectivity in *Anopheles arabiensis* colony mosquitoes fed on blood from patients infected with *Plasmodium vivax.* Mosquitoes were tested at six post-infection time points (days 0.5, 1, 6, 9, 12, 15). The head and thorax and the abdomen for each specimen were tested separately with each method. Agreement between methods at each infection stage was measured using Cohen’s kappa measure of test association.

**Results:**

Infection status of mosquitoes was assessed in approximately 90 head/thorax and 90 abdomen segments at each time point; in total, 538 head/thorax and 534 abdomen segments were tested. In mosquitoes bisected after 0.5, 1, and 6 days post-infection (dpi), the mt *COX-I* PCR detected *Plasmodium* DNA in both the abdomen (88, 78, and 67%, respectively) and head/thorax segments (69, 60, and 44%, respectively), whilst CSP ELISA detected sporozoites in only one abdomen on day 6 post-infection. PCR was also more sensitive than ELISA for detection of *Plasmodium* in mosquitoes bisected after 9, 12, and 15 dpi in both the head and thorax and abdomen. There was fair agreement between methods for time points 9–15 dpi (κ = 0.312, 95% CI: 0.230–0.394).

**Conclusions:**

The mt COX-I PCR is a highly sensitive, robust method for detecting *Plasmodium* DNA in mosquitoes, but its limited *Plasmodium* life-stage specificity cannot be overcome by bisection of the head and thorax from the abdomen prior to PCR. Thus, the mt COX-I PCR is a poor candidate for identifying infectious mosquitoes.

**Graphical Abstract:**

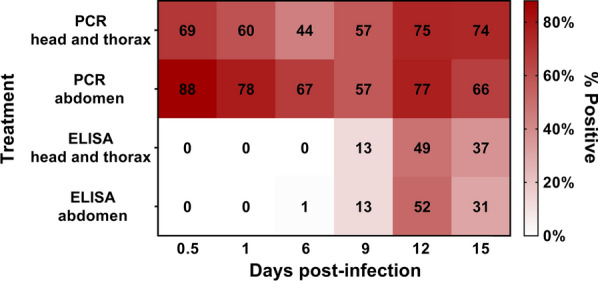

## Background

*Plasmodium* sporozoites are injected into a new host with mosquito saliva during the bite of an infectious mosquito. Prior to becoming infectious, a mosquito must ingest the sexual blood stage (gametocytes) of *Plasmodium* parasites from a human host during blood-feeding. This begins the sporogonic development cycle of *Plasmodium* within the mosquito. The timeline for sporogony varies by *Plasmodium* species, and *P. vivax* has the shortest cycle of human malaria parasites [1]. Within the mosquito abdomen, sporogonic development begins with gametogenesis and the formation of a zygote (within 48 h post-infection), to an ookinete (16–32 h post-infection) and an oocyst (6–9 days post-infection [dpi]), before developing into sporozoites (9–14 dpi). These sporozoites rupture from the abdominal oocyst and migrate through the hemolymph and the thorax to the salivary glands—at this point the mosquito is considered to be infectious [1]*.* Given how few sporozoites may be egested during blood feeding and result in parasite transmission, the bite of a mosquito with any number of sporozoites in its salivary glands is considered to be infectious [2].

When characterizing malaria epidemiology and intervention effectiveness, an important entomological factor to consider is the percentage of mosquitoes that are infectious, also known as the sporozoite rate [3, 4]. Taken together, the sporozoite and human biting rates enable estimation of the frequency of infective bites by *Anopheles* mosquitoes over time—known as the entomological inoculation rate (EIR). EIR remains an important metric to quantify malaria parasite transmission and the most widely used measure for understanding vector control effectiveness [4]. In settings where transmission occurs, sporozoite detection can incriminate malaria vectors and reveal those species that are driving *Plasmodium* transmission [5]. Historically, microscopy has been used to identify sporozoites in the salivary glands for calculation of sporozoite rates, but this method is technically challenging given the time and skill required to dissect a high volume of specimens, while also not allowing the identification of *Plasmodium* species [6]. Given the challenges of microscopy and the significance of sporozoite rates to understanding parasite transmission, it is important that methods of detecting sporozoites are both specific and sensitive.

 Circumsporozoite (CSP) enzyme-linked immunosorbent assays (ELISA) have been considered the “gold standard for vector incrimination” since their development in 1984 [7, 8]. In addition to overcoming the technical limitations of salivary gland dissection and microscopy, the CSP ELISA specifically detects the circumsporozoite protein expressed only by sporozoites and enables *P. falciparum* and *P. vivax* species determination. However, it has garnered criticism for being less sensitive than microscopy, particularly when fewer than 100 sporozoites are present in the salivary glands [7, 9]. While specific to sporozoites, CSP ELISA can detect sporozoites still developing in the midgut oocyst of the mosquito abdomen, prior to reaching the salivary glands when the mosquito is considered infectious*.* High rates of false positives due to cross-reactivity with non-*Plasmodium* antigens have also been observed [10]. A study in which positive ELISA results were subsequently analyzed by *Plasmodium*-specific polymerase chain reaction (PCR) targeting the 18 s-rRNA, found that a high frequency of ELISA false positives can result from zoophilic vectors where an unidentified heat-labile antigen from animal blood is cross-reactive [10]. These limitations have drawn attention to the need for more sensitive and specific methods of vector incrimination. In areas with widespread vector control and reduced endemicity nearing elimination, shifts in vector species composition and behavior can occur [11, 12]. Opportunistic vectors may replace primary or more anthropophilic vector species; thus their contribution to disease transmission may be misrepresented by ELISA alone, where an unknown blood meal source may contribute to false positives for sporozoites, potentially misinforming downstream decisions regarding vector control.

PCR methods for *Plasmodium* detection have been considered too complex and expensive for field use. A one-step PCR method developed by Echeverry et al., which targets the mitochondrial cytochrome oxidase I (*COX-I*) gene of *Plasmodium*, was designed as a streamlined, sensitive, and cost-effective alternative to the commonly used 18 s-rRNA nested PCR [13]; this approach has also been considered as a way to overcome the limited sensitivity of the CSP ELISA, detecting *Plasmodium* spp. DNA from as few as two parasites [14]. Despite the increased sensitivity, the mt COX-I PCR detects DNA that is present in all life stages of the parasite, thus lacking the specificity demonstrated by the detection of expressed protein with the CSP-specific ELISA [14]. This limitation is possibly overcome through the bisection of the specimen anterior to the naturally occurring break point between the abdomen and thorax. Using the nested PCR for *Plasmodium* DNA detection, fewer false positives were observed from the most anterior bisection point, between the second and third legs [15]. It is also possible that despite bisection, PCR-based methods may detect the DNA of any parasite stage that remains in the upper digestive tract of the mosquito following an infected blood meal, or those that are circulating within the mosquito hemocoel outside of the salivary glands. However, the latter can also be argued as a limitation of the CSP ELISA [15].

The aim of this study was to determine whether PCR using the one-step mt COX-I PCR method [14] could provide the same or better information as CSP ELISA towards detecting mosquitoes with salivary gland sporozoites, considering the importance of bisection to reduce false positives in time points prior to sporozoite development.

## Methods

### Sample collection

Five patients, self-presenting to the Adama, Ethiopia malaria clinic, were asked to donate a venous blood sample (5 ml) in lithium heparin tubes (BD Vacutainer^®^). Written informed consent was obtained from blood donors and/or guardians in the case of minors. Asexual parasite and gametocyte densities were quantified by two expert microscopists on thick blood films prepared from finger-prick blood samples, screening against 1000 leukocytes.

Membrane feeding was performed in two groups. Infected treatment groups were mosquitoes fed on blood from *P. vivax* parasite carriers, while the control group consisted of mosquitoes fed on blood from a clinically and microscopy-confirmed *Plasmodium*-negative blood sample.

### Mosquito infection

Blood from patients with microscopy-confirmed *Plasmodium vivax* infections was fed to locally reared 3–5-day-old female *Anopheles arabiensis* field-colonized mosquitoes using a membrane feeding apparatus. Female mosquitoes (*n* = 280) were offered fresh patient blood in water-jacketed glass feeders (mini-feeder; Coelen Glastechniek, Arnemuiden, the Netherlands) that were covered with an artificial membrane (PARAFILM^®^, Sigma-Aldrich) and connected to a circulating water bath (JULABO GmbH; Seelbach, Germany) maintained at 39 °C. Female mosquitoes were starved for 12 h and put in net-covered paper cups (Laan, Heiloo) containing 40 mosquitoes each and fed in the dark for 25 min. Fully fed mosquitoes were transferred to clean, custom-made (25 cm × 25 cm × 25 cm) cages 20 min after feeding. Mosquitoes were reared and maintained to time points after infectious blood feeding at controlled temperature (26 ± 3 °C) and relative humidity (70 ± 10%) and maintained with 10% sucrose solution ad libitum. Ninety mosquitoes from the same colony, fed blood from an individual without *Plasmodium* infection, served as negative controls.

### Mosquito processing

For each experimental infection, 30 mosquitoes were killed in a –20 °C freezer at each time point—12 h, and 1, 6, 9, 12, and 15 days post-feeding—and stored on desiccant at −20 °C until processing. Three experimental infections were conducted to produce biological replicates and increase sample size for subsequent molecular work.

Control mosquitoes were collected following a similar workflow as described above. Uninfected, human blood was used to feed control mosquitoes, with only one blood-feeding event that yielded 15 mosquitoes for each time point. These specimens were tested as controls for PCR and ELISA experiments and 20 for midgut dissections.

Seven days following feeding, an additional 20 mosquitoes from each infection event were dissected, and oocyst presence was assessed microscopically after staining with 1.0% Mercurochrome (Sigma-Aldrich, Taufkirchen, Germany) to determine the success of each infection. Experimental mosquito specimens were bisected using a sterilized scalpel, which was dipped in pure ethanol and wiped clean with a tissue between each use. Mosquitoes were bisected between the second and third legs—anterior to the naturally occurring break point between thorax and abdomen [15]. Any additional body parts that remained intact following bisection (i.e., legs) were included with the associated body segment. Mosquito heads and thoraces, and separately abdomens, were completely homogenized with pestles in 100 μl of molecular-grade water. Fifty microliters of homogenate of the same specimen was used for DNA extraction and the remaining homogenate used for the CSP ELISA assay. DNA was extracted from the homogenate following the cetyltrimethyl ammonium bromide (CTAB) method [14], using 155 μl of 2% CTAB extraction buffer. Mosquito triturate was prepared by mixing homogenate with grinding buffer (blocking buffer plus 0.5% Igepal CA-630), outlined in the MR4 protocol [16].

### CSP ELISA

CSP ELISA for the detection of *P. vivax* vk 210 parasites was conducted on head/thorax and abdomen segments of specimens according to the Malaria Research and Reference Reagent Resource Center (MR4) protocol, using monoclonal antibodies (mAbs) and positive control from BEI Resources (cat. no. MRA-1028 K) [16]. In short, the 96-well plate (Corning; 3366) was coated with 50 µl of 2 µg/ml in 10 mM phosphate-buffered saline (PBS) *P. vivax* vk 210 capture mAb for 30 min and then replaced with 200 µl blocking buffer (0.5% w/v casein, 0.0002% w/v phenol red in 10 mM PBS, pH 7.4) for 1 h [9]. Following a 2-h incubation of mosquito triturate (50 µl), after removing the blocking buffer, wells were washed twice. Subsequently, plates were incubated with 50 µl of *P. vivax* 210 detection mAb (1 µg/ml in blocking buffer) for 1 h and washed three times before incubation for 30 min in the dark after adding 100 µl 2,2'-azino-bis(3-ethylbenzothiazoline-6-sulphonic acid) (ABTS) substrate solution (SeraCare; 5120–0032). The optical density of each sample was measured using the EMax Plus Microplate Reader (Molecular Devices, LLC) and positives calculated above two times the mean absorbance values of the negative controls. Positive recombinant controls and negative control *An. arabiensis* colony mosquitoes (AHRI) were tested in addition to experimental specimens in each plate.

### Mt COX-I PCR

*Plasmodium* DNA in the head/thorax and abdomen of specimens was amplified using modified “fast COX-I” PCR methods [14]. The *COX-I* region of the *Plasmodium* mitochondrial DNA was amplified using the AccuStart II PCR ToughMix (Quantabio, 95142) with 0.8 mM COX-IF and COX-IR primers and 3 μl of template for each 20 μl reaction. Quantified, extracted DNA of *P. falciparum* from culture (0.12 ng/µl) was used as a positive reaction control, given that PCR amplification is not *Plasmodium* species-specific, and parasite DNA cannot be quantified independently of mosquito DNA following extraction [14]. Thermocycling was conducted in a T100 thermal cycler (Bio-Rad, 1861096) under the following conditions: 94 °C for 3 min; 40 cycles of 94 °C for 30 s, 65 °C for 1 min, 72 °C for 1 min; with no final extension. PCR amplification was confirmed by electrophoresis using 1% agarose gel. Positive results were determined by expected band size at approximately 520 bp.

### 18S small subunit ribosomal ribonucleic acid (SSU rRNA) nested PCR

Following the results observed from the mt COX-I PCR, the nuclear 18S SSU-rRNA nested PCR was conducted on a subset of specimens to investigate whether concordance between methods could be improved using the less sensitive nested PCR method. Lack of available samples prevented additional experimentation on mosquitoes from all three infections. *Plasmodium* DNA in the head/thorax and abdomen of 90 specimens from a single infection (Infection 3) were amplified by nested PCR reactions [13] for comparison to the mt COX-I PCR and CSP ELISA. The nuclear SSU-rRNA gene was amplified first (nest 1) using 0.75 U recombinant Taq polymerase (Invitrogen, 10342053) with 1× PCR buffer, 80 μM dNTPS, 0.8 mM MgCl_2_, and 2 μM of rPLU1 and rPLU5 primers, and 1 μl of DNA template for a 10 μl reaction. Thermocycling was completed under the following conditions: 94 °C for 4 min; 35 cycles of 94 °C for 30 s, 55 °C for 1 min, 72 °C for 1 min; 72 °C for 4 min final extension. One microliter of the completed nest 1 PCR reaction was used as template in the following genus-specific nest 2 PCR reaction. Nest 2 followed the same conditions as nest 1, but instead used rPLU3 and rPLU4 primers with an annealing temperature of 62 °C. Results were visualized on a 1% agarose gel, whereby positives were determined by amplification of target DNA near 240 base pairs.

### Analyses

The one-step mt COX-I PCR and CSP ELISA experiments were used for the detection of *Plasmodium* in the two body segments at six different time points following infectious feed. Agreement between the results of the mt COX-I PCR, 18S SSU-rRNA nested PCR, and the CSP ELISA was measured using Cohen’s kappa measure of test association [17]. The significance of the discordance between individuals was determined using the McNemar test. A logistic regression was used to compare the probability of positive outcomes based upon dpi, body part, and parasite density of the blood sample. For CSP ELISA regression, parasite density was not included as a variable, given that the non-sporozoite life stages reported in Table 1 would be undetected. Additional Cochran-Armitage tests for trend were conducted on subsets of data to determine significant trends between each independent variable and positive outcomes (IBM SPSS Statistics 26, IBM Corp., Armonk, NY, USA).

**Table 1:** Summary statistics for blood samples and midgut dissection, relative to each independent infection event

Infection number	Blood microscopy result	Asexual stage density	Gametocyte density	No. midguts dissected	No. mosquitoes infected (microscopy)	% Infected
1	*P. vivax*	9233/μl	199/μl	5	1	20%
2	*P. vivax*	8627/μl	980/μl	20	0	0%
3	*P. vivax*	5892/μl	897/μl	20	6	30%

*Plasmodium* parasite density of human blood samples used for each infection and subsequent confirmation of mosquito infection by midgut oocyst dissection at 7 dpi

### Ethical considerations

The study protocol was reviewed and approved by institutional ethics review boards of the Armauer Hansen Research Institute (AAERC, P035/17), the University of Notre Dame (19–09-5517), and the National Research Ethics Review Committee of Ethiopia (310/150/2018).

## Results

A total of 650 mosquitoes were successfully blood-fed, maintained to six post-infection experimental time points, and each analyzed for infectivity by PCR and ELISA. Two of the three patients with microscopically detectable trophozoites and gametocytes infected at least one mosquito as confirmed with oocyst detection by microscopy 7 dpi as indicated in Table 1. Only five mosquitoes were dissected for the first infection due to scarcity of mosquitoes. Control mosquitoes (*n* = 110) fed from uninfected human blood developed no *Plasmodium* parasites detected by microscopy or experimental methods and were therefore excluded from statistical analysis.

### Mt COX-I PCR results

For 0.5, 1, and 6 dpi, 57% (*n* = 156/270) of head/thorax and 77% (208/269) of abdomen segments tested positive for *Plasmodium* DNA by PCR (Table 2). The proportion of mosquitoes positive for *Plasmodium* DNA declined from 12 h to 6 dpi for both the head/thorax (69–44%) and the abdomen (88–67%, *P* < 0.001). During bisection, blood was observed by microscopy in the thorax of mosquitoes at both 0.5- and 1-day time points. For time points 9, 12, and 15 dpi, 69% (185/268) of head/thorax and 66% (176/265) of abdomen segments tested positive by the mt COX-I PCR (Table 2). The proportion of positive mosquitoes was higher for days 12 and 15 than day 9 for both body segments, an increase of up to 18% (66–75 to 57%, *P* = 0.011). A logistic regression showed overall statistically significant differences (*χ*^2^ = 46.004, *df* = 7, *P* < 0.001) between the effects of parasite density, time post-infection, and body part on the likelihood that mosquitoes (head/thorax and abdomen) tested positive for *Plasmodium*. The model explained 1.7% (Nagelkerke *R*^2^) of the variance in positive results and correctly classified 67.6% of cases. Earlier time points (0.5, 1, and 6 dpi) were associated with an increased likelihood of becoming PCR-positive, and while a positive association was observed in the model between infection and parasite density, its contribution was not considered statistically significant (*P* = 0.054).

**Table 2:** Summary of mt COX-I PCR and CSP ELISA results

Days post-infection	Theoretical infection stage	Head/thorax % (*n*/*N*)		Abdomen % (*n*/*N*)	
		COX-I PCR	CSP ELISA	COX-I PCR	CSP ELISA
0.5	Macrogametocytes	69 (62/90)	0 (0/90)	88 (79/90)	0 (0/90)
1	Macrogametocytes	60 (54/90)	0 (0/90)	78 (69/89)	0 (0/90)
6	Oocysts only	44 (40/90)	0 (0/90)	67 (60/90)	1 (1/89)
9	Oocysts and Sporozoites	57 (51/89)	13 (12/89)	57 (51/89)	13 (12/89)
12	Oocysts and Sporozoites	75 (67/89)	49 (44/89)	77 (66/86)	52 (47/88)
15	Sporozoites	74 (67/90)	37 (33/90)	66 (59/90)	31 (28/90)

Number (*N*) of specimens and percentages of *Plasmodium vivax*-positive results by PCR and ELISA based upon anterior (head and thorax) and posterior (abdomen) body segments of *Anopheles arabiensis* mosquitoes

### CSP ELISA results

CSP ELISAs conducted on early infection stages (0.5–6 dpi) were overwhelmingly negative in the head and thorax segments (Table 2). Only one abdomen was found positive on day 6 post-infection, for which the associated head and thorax remained negative. Between 6 and 12 dpi, positives were detected in equal proportions (33%) in both the head/thorax and abdomen segments, significantly more than 0.5–6 dpi (*P* < 0.001). More body segments tested positive at day 12 post-infection. A logistic regression was used to investigate the effects of time post-infection and body part on the likelihood that mosquitoes tested positive for CSP protein. Differences between time post-infection and body segment were statistically significant, *χ*^2^ = 40.496, *df* = 8, *P* < 0.001. The logistic regression model explained 34.9% (Nagelkerke *R*^2^) of the variance in positive results and correctly classified 81.4% of cases. The later post-infection time points were associated with an increased likelihood of becoming CSP ELISA-positive, though body section did not contribute significantly to positive CSP ELISA outcomes.

### Agreement between the mt COX-I PCR and CSP ELISA outcomes

The mt COX-I PCR consistently detected *Plasmodium* DNA in samples from 0.5 to 6 dpi, as indicated by CSP protein that was not detected by the CSP ELISA until time points following 6 dpi. For 0.5–6 dpi, the mt COX-I PCR and CSP ELISA did not demonstrate agreement between positive outcomes of the two methods (*κ* = 0.00) in the head/thorax. However, agreement between methods improved to “fair” for mosquitoes bisected 9–15 dpi in the head and thorax (*κ* = 0.312, 95% CI: 0.230–0.394).

### Discordance between mt COX-I PCR and CSP ELISA outcomes in individual specimens

The greatest discordance between methods was observed in mosquitoes bisected 0.5–6 dpi, when PCR-positive/ELISA-negatives accounted for 58 and 77% of head/thorax and abdomen segments, respectively (Table 3). However, discordance where ELISA-positive tested negative by PCR was ≤ 5% for both body segments at all time points. Estimations of infectiousness using the two methods were significantly different for body segments bisected 0.5, 1, and 6 dpi and 9, 12, and 15 dpi (McNemar: *P* < 0.001).

**Table 3:** Concordant and discordant mt COX-I PCR results

Infection stage	Body part	COX-I PCR + ELISA + (concordant)	COX-I PCR + ELISA − (discordant)	COX-I PCR − ELISA + (discordant)	COX-I PCR − ELISA − (concordant)
0.5, 1, 6 dpi	Head and thorax	0 (0/270)	58 (156/270)	0 (0/270)	42 (114/270)
	Abdomen	0.4 (1/ 269)	77 (207/269)	0 (0/269)	22 (61/269)
9, 12, 15 dpi	Head and thorax	31 (84/268)	37 (101/268)	2 (5/268)	29 (78/268)
	Abdomen	30 (79/265)	37 (97/265)	2 (6/265)	31 (83/265)

Percentages (% (n/N)) of concordant and discordant mt COX-I PCR results compared to the CSP ELISA, by infection stage and body segment

### Agreement between nested PCR and ELISA outcomes

For 0.5 to 6 dpi, 31% of head/thorax and 60% of abdomen segments tested positive for *Plasmodium* DNA, for which there were zero CSP ELISA positives (Table 4). When compared between the head and thorax for these post-infection time points, the nested PCR and CSP ELISA showed poor agreement between outcomes of the two methods (*κ* = 0.00). For 9–15 dpi, 63% of head/thorax and 73% of abdomen segments tested positive for *Plasmodium* DNA by nested PCR (Table 4). Nearly 50% of those positives were concordant with the CSP ELISA results for the same specimen (Table 4). Agreement between the nested PCR and ELISA methods improved to “substantial” agreement in 9–15 dpi stages (*κ* = 0.644, 95% CI: 0.492–0.796). When kappa values between mt COX-I and nested PCR were compared, Cohen’s kappa was higher by only 0.066 when nested PCR was used and results compared within the third infection. When the mt COX-I for all infections and nested PCR for the third infection were compared, agreement was higher by 0.332.

**Table 4:** Concordant and discordant results of the nested PCR

Infection Stage	Body part	Nested PCR + ELISA + (concordant)	Nested PCR + ELISA − (discordant)	Nested PCR − ELISA + (discordant)	Nested PCR − ELISA − (concordant)
0.5, 1, 6 dpi	Head and thorax	0 (0/90)	31 (28/90)	0 (0/90)	69 (62/90)
	Abdomen	0 (0/89)	60 (53/89)	0 (0/89)	40 (36/89)
9, 12, 15 dpi	Head and thorax	48 (43/90)	16 (14/90)	2 (2/90)	34 (31/90)
	Abdomen	47 (41/88)	27 (24/88)	2 (2/88)	24 (21/88)

Percentages (% (n/N)) of concordant and discordant 18sr-RNA nested PCR results for a single infection compared to the CSP ELISA, by infection stage and body segment

## Discussion

In this study, we compared the mitochondrial COX-I PCR with CSP ELISA throughout *Plasmodium* ingestion and invasion, following bisection. *Plasmodium* DNA was detected by PCR in bisected head/thorax segments during all time points, although abdomens showed a slightly higher rate of PCR positivity before development. CSP ELISA alone detected fewer positive specimens overall, and all but one positive occurred after 6 dpi. However, these positives were observed in nearly equal proportions between body sections. When compared directly, the mt COX-I PCR and CSP ELISA results improved from “poor” to “fair” agreement in the head and thorax for mosquitoes bisected 9, 12, and 15 dpi (from *κ* = 0.000 to 0.312). The use of a less sensitive, nuclear nested PCR approach improved post-development agreement with ELISA to “substantial” (*κ* = 0.644).

This study expanded on a previous study, in which Foley et al*.* [15] investigated the role of bisection in understanding PCR-derived estimates of *P. falciparum* sporozoite rates. They concluded that bisection between the second and third legs is a critical first step in preventing false positives by PCR from non-sporozoite life stages. When Foley et al*.* [15] used the nuclear 18S SSU-rRNA nested PCR method to test the head and thorax following this bisection method, zero false positives were observed 6–7 dpi [18]. When applied to our study using *P. vivax*, bisection between the second and third legs was found to limit false positives by CSP ELISA from sporozoites within abdominal oocysts, but did not reduce the number of positives by the mt COX-I PCR in the head and thorax at any stage of infection. Rather, the greatest number of positives was observed at 0.5 and 1 day post-infection, a time point not included in Foley et al*.* [15]*.* While the number of positives was reduced using the nested PCR, it was not reduced to zero for head/thorax segments at any stage and still demonstrated discordance in time points 9–15 dpi when compared to CSP ELISA. Given bisection practices, a subsequent experiment did not indicate carryover from the scalpel as a source of contamination (Hendershot, unpublished).

**Fig. 1:**
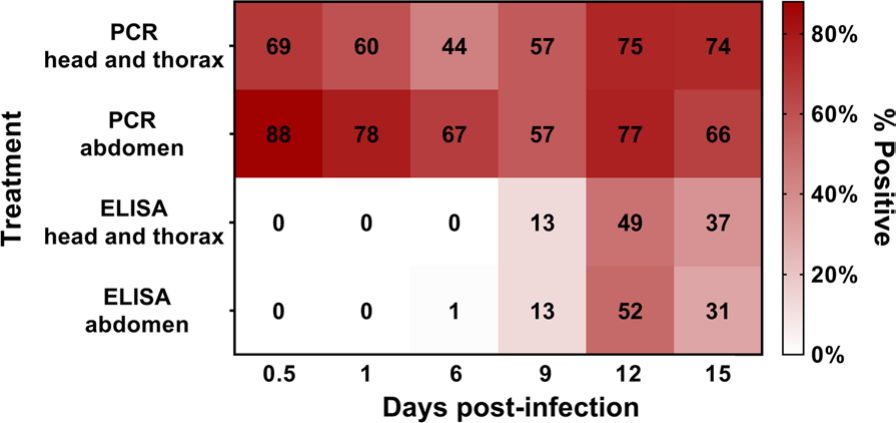
Heat map of positive results by mt COX-I PCR and CSP ELISA. Percentage of positive body segments for mt COX-I PCR and CSP ELISA at each of the six time points of infection whereby the intensity of color (red) indicates a higher percentage of positive results by the respective methods. *COX-I* cytochrome oxidase I, *PCR* polymerase chain reaction, *CSP ELISA* circumsporozoite enzyme-linked immunosorbent assay

The greatest discordance between CSP ELISA and the mt COX-I PCR was observed at time points immediately following an infective blood meal (0.5 and 1 day), when infectious sporozoites would not have had time to develop (Fig. 1). The presence of blood observed in both body segments following bisection suggests incomplete migration of the blood to the midgut. Bisection presented the opportunity for blood leakage during dissection [19] and the potential for carryover contamination from one sample to another within samples for a specific blood feeding and time point, though additional experimentation indicted that carryover contamination was an unlikely cause of positives. Previously, it has been assumed that the blood that remains in the mosquito pharynx is insufficient to result in a false-positive reaction by ELISA from the blood meal source [19]. However, this cannot be assumed of the mt COX-I PCR, where as few as two parasites can give a positive result [14]. Therefore, the sustained presence of trophozoites or gametocytes in the human blood meal likely contributed to positive results by PCR in early time points. Parasite densities of the patients in this study were considered high; therefore, outcomes might differ if blood with lower parasite density was fed to mosquitoes, considering the small volume ingested by mosquitoes during blood feeding. Though this study was not designed to test parasite density given the limited sample size, it was not a significant predictor in the associated regression model.

For mosquitoes bisected from 9 to 15 dpi, the mt COX-I PCR detected twice as much *Plasmodium* as CSP ELISA in the head and thorax, resulting in statistically poor agreement between these methods. Agreement between PCR and CSP ELISA results increased (*k* = 0.644) when the nuclear 18S SSU-rRNA nested PCR method was used—an observation corroborated by findings from Stone et al*.*, where concordance was found to be as high as 85% in late-stage infections [20]. The differences in agreement of the mt COX-I PCR and nuclear nested PCR with CSP ELISA may be explained by the limited experimental sample size for the nested PCR, given the slight difference in kappa values between PCR assays for the third infection. However, it is also likely a reflection of the variable limits of detection, whereby nuclear nested PCR and CSP ELISA are more comparable (6 parasites/1 µl and 125–230 sporozoites/30 µl, respectively) [13, 21, 22]. Additional PCR positives found using the mt COX-I PCR may be attributed to the increased sensitivity of the assay, which was shown to be > 460-fold as sensitive as the nested PCR method [14].

As PCR has become standard in many laboratories, it has been used as an alternative method to the CSP ELISA [23–27]. In Kenya, PCR-derived sporozoite rates as high as 9% in species within the *An. funestus* complex have prompted additional research into their role as vectors and have been used to inform vector control efforts [27, 28]. However, this dataset demonstrates the limited application of PCR for sporozoite (or more accurately “infection”) detection by the disagreement observed in results at early time points following an infected blood meal. Even following sporozoite development, positives may arise from sporozoites located outside of the salivary glands, in other mosquito tissues and/or present in the hemocoel [9]. This cannot be overcome by bisection between the second and third legs before PCR or ELISA, and thus the overestimated “infection” rate would be relative to the sensitivity reported by each assay—for which the mt COX-I PCR has been shown to be highly sensitive [14]. However, the benefit of PCR-based methods remains the ability to detect all human *Plasmodium—*a limitation of the CSP ELISA, which can currently only detect *P. falciparum* and *P. vivax.*

The use of human blood samples naturally infected with *P. vivax* in mosquito-feeding assays allowed us to mimic natural infection events as they would occur in nature, making results of this study pertinent to the interpretation of data from field-collected *Anopheles* [29] using these methods for *Plasmodium* detection*.* The collection method of wild *Anopheles* and site-specific endemicity are important considerations in the interpretation of PCR-derived infection rates. Based on the results presented, positives observed by PCR prior to sporozoite development are strongly indicative of which mosquitoes are taking an infected blood meal. Therefore, data from sampling biased towards recently fed mosquitoes would overestimate PCR-derived *Plasmodium* infection rates. *Plasmodium* species detected by PCR would be more representative of the diversity of *Plasmodium* infections in the local human population, rather than a reflection of the diversity of *Plasmodium* transmitted by local vector species. Residual parasite DNA from a blood meal cannot be ruled out entirely or distinguished from truly infectious mosquitoes. Thus, sampling biased towards anthropophagic mosquitoes, such as human landing collections (HLC), may overrepresent infection rates given that some *Anopheles* have been documented to engage in human host-seeking behaviors even following a recent blood meal [30]*.* For example, when the mt *COX-I* was previously applied in Echeverry et al*.* [14] to samples collected by HLC at a low-endemicity area of the Solomon Islands, fewer mosquitoes tested positive for *Plasmodium* parasites and their abdominal status was not recorded. Therefore, PCR-derived infection rates should be assumed to be overestimates of actual sporozoite rates.

While the detection of *Plasmodium* DNA by PCR and CSP protein by ELISA has been shown to overestimate the number of infectious mosquitoes [9, 10, 15, 31, 32], it is not possible to distinguish truly infectious mosquitoes within our study without salivary gland dissection of each specimen. Moreover, contaminants among parts of the same specimen or between specimens may be overrepresented by results of the highly sensitive mt COX-I PCR. Given that the logistic regression could only account for 1.5% of variance between PCR outcomes, additional factors apart from time post-infection, parasite density, and body segment impacted the data—as well as a limited number of experiments that informed the logistic regression. Other PCR methods for detecting *Plasmodium* DNA in mosquitoes were considered, as they improve upon limitations of sensitivity, specificity, or rigor. Quantitative PCR (qPCR), reverse transcription real-time PCR (RT-PCR), and TaqMan assays improve upon the sensitivity of the gold-standard nested PCR approach, while also utilizing probes specific to sporozoites [33–35]. Due to practical limitations that exist in their scalable application to vector control operations, including sample size, specimen storage, and time and resource costs, they were excluded from this study. Future studies would benefit from including salivary gland dissection to microscopically confirm infectious sporozoites and the addition of a qPCR method to distinguish low-density sporozoite infections from non-sporozoite positives from the mt COX-I PCR.

Based upon our data, results stemming from the use of PCR alone for *Plasmodium* detection should be interpreted with caution. It can be recommended that for the detection of infectious sporozoites, a protocol where bisection between the second and third legs prior to CSP ELISA can help to minimize false positives that may be due to inclusion of abdomen potentially containing blood meal with cross-reactive proteins or abdominal oocysts. Following initial CSP ELISA testing, boiling of any positive samples for retesting by CSP will continue to help minimize false positives that may be due to residual cross-reactive proteins from blood in the head and thorax or other unidentified sources [10]. An additional step of PCR can be added for increased confidence in positive confirmation. When additional PCR methods, such as mosquito species identification or insecticide resistance, are of equal consideration for the same specimen, homogenization in a neutral solution (such as water) can be conducted first and homogenate divided between methods for DNA and protein detection with success, given the difficulty of successful DNA extraction and PCR amplification from ELISA homogenate.

## Conclusion

Results indicate that the PCR is a robust method for detecting *Plasmodium* DNA within a mosquito, but its limited *Plasmodium* life-stage specificity makes it a poor candidate for detecting *infectious* mosquitoes. The “gold-standard” CSP ELISA should not be replaced by PCR-based methods for detecting infectious *Plasmodium* but can be used in conjunction with CSP ELISA for the confirmation of positive infectious mosquitoes and detection of low-density infections within a mosquito.

## Data Availability

Data supporting the conclusions and outcomes of this article are included within the article. The raw datasets presented and analyzed in this study are available upon request from the corresponding author.

## Abbreviations

EIR: Entomological inoculation rate
CSP ELISA: Circumsporozoite enzyme-linked immunosorbent assay
Mt: Mitochondrial
*COX-I*: Cytochrome oxidase I
PCR: Polymerase chain reaction
Dpi: Days post-infection
HT: Head and thorax
ABD: Abdomen
CTAB: Cetyltrimethyl ammonium bromide
SSU rRNA: Small subunit ribosomal ribonucleic acid
PBS: Phosphate-buffered saline
PBS-T: Phosphate-buffered saline–0.05% Tween 20
